# Optical Characterization of Two-Layered Turbid Media for Non-Invasive, Absolute Oximetry in Cerebral and Extracerebral Tissue

**DOI:** 10.1371/journal.pone.0064095

**Published:** 2013-05-21

**Authors:** Bertan Hallacoglu, Angelo Sassaroli, Sergio Fantini

**Affiliations:** Department of Biomedical Engineering, Tufts University, Medford, Massachusetts, United States of America; Tokyo Metropolitan Institute of Medical Science, Japan

## Abstract

We introduce a multi-distance, frequency-domain, near-infrared spectroscopy (NIRS) method to measure the optical coefficients of two-layered media and the thickness of the top layer from diffuse reflectance measurements. This method features a direct solution based on diffusion theory and an inversion procedure based on the Levenberg-Marquardt algorithm. We have validated our method through Monte Carlo simulations, experiments on tissue-like phantoms, and measurements on the forehead of three human subjects. The Monte Carlo simulations and phantom measurements have shown that, in ideal two-layered samples, our method accurately recovers the top layer thickness (*L*), the absorption coefficient (µ*_a_*) and the reduced scattering coefficient (**µ**′*_s_*) of both layers with deviations that are typically less than 10% for all parameters. Our method is aimed at absolute measurements of hemoglobin concentration and saturation in cerebral and extracerebral tissue of adult human subjects, where the top layer (layer 1) represents extracerebral tissue (scalp, skull, dura mater, subarachnoid space, etc.) and the bottom layer (layer 2) represents cerebral tissue. Human subject measurements have shown a significantly greater total hemoglobin concentration in cerebral tissue (82±14 µM) with respect to extracerebral tissue (30±7 µM). By contrast, there was no significant difference between the hemoglobin saturation measured in cerebral tissue (56%±10%) and extracerebral tissue (62%±6%). To our knowledge, this is the first time that an inversion procedure in the frequency domain with six unknown parameters with no other prior knowledge is used for the retrieval of the optical coefficients and top layer thickness with high accuracy on two-layered media. Our absolute measurements of cerebral hemoglobin concentration and saturation are based on the discrimination of extracerebral and cerebral tissue layers, and they can enhance the impact of NIRS for cerebral hemodynamics and oxygenation assessment both in the research arena and clinical practice.

## Introduction

The ability to noninvasively measure the concentrations of oxidized and reduced hemoglobin (i.e. oxy-hemoglobin and deoxy-hemoglobin, respectively) in biological tissues with high temporal resolution (in the order of milliseconds) is a prominent feature of near-infrared spectroscopy (NIRS) technology. This in turn allows for real-time monitoring and spatial mapping of underlying physiological parameters such as blood volume, blood perfusion, metabolic rate of oxygen, and oxygen delivery. Of current interest is the *absolute* measurement of the concentration and oxygen saturation of hemoglobin in the human brain, particularly by taking into account the extracerebral tissue contributions that can confound non-invasive optical measurements.

Over the past years, NIRS has seen an increasing appeal in the clinical realm. This has given rise to commercial devices that perform non-invasive cerebral oximetry by providing an estimate of oxygen saturation of hemoglobin in the human head (variously termed tissue oxygenation index (TOI), tissue saturation index (TSI), regional oxygen saturation (rSO2) and oxygen saturation (SO2)) (for a review, see Wolf *et al.* 2007 [1]). Currently explored applications of clinical relevance include monitoring post traumatic brain injury [2], ischemic stroke [3], coronary bypass surgery [4], and detecting, for instance, cardiopulmonary dysfunction [5], cerebral hemorrhage [6], and impaired cerebral autoregulation [7]. Despite the strong indication that accurate real-time NIRS measurement of cerebral hemoglobin parameters could significantly improve critical patient care and routine clinical practice [2], [8], [9], concerns remain on the reliability of the current methods particularly due to extracerebral tissue contamination in the NIRS signals [10], [11].

In a common research paradigm, functional NIRS (fNIRS) approaches are employed to study hemodynamic and metabolic responses to brain activation, which involve measurements of relative changes of oxy- and deoxy-hemoglobin concentrations [12], [13], [14]. Existing fNIRS strategies to partly account for extracerebral hemodynamic trends include devising task-based experimental protocols to inhibit systemic contaminants [15], [16], [17], [18]; averaging measured hemodynamic changes over multiple events (as employed by most fNIRS studies) [19]; and performing two-distance measurements and fitting and regressing out detected near signals (i.e. near the point of illumination) from far signals (i.e. far from the point of illumination) [19], [20], [21], [22], [23]. Other strategies employ methods such as adaptive filtering [24], [25]; principal component analysis (PCA) [26], [27], [28]; and independent component analysis (ICA) [28], [29]. Furthermore, computationally more sophisticated diffuse optical tomography (DOT) methods feature volumetric mesh based modeling of the human head with the aim of localizing measured hemodynamic changes under the assumption that underlying tissue is homogenous [30], [31], [32], [33] or layered [34].

While addressing the extracerebral contamination to some degree, fNIRS studies do not perform a baseline correction of measured dynamic changes (i.e. measured changes in optical coefficients divided by baseline/resting state optical coefficients), which has been shown to be an essential step for the determination of the true inter-individual variability in a cohort in rat [35]. DOT studies recognize the importance of baseline values and employ absolute baseline/resting optical coefficients in their forward modeling [30], [34]; however these studies rely on assumed values from the literature, thus neglecting inter-subject and intra-subject variability in these parameters. Relative measurements such as those typically employed in fNIRS lack the ability to provide information about the baseline state of the brain that result from baseline cerebral blood flow, blood volume, and metabolic rate of oxygen. Absolute NIRS measurements of the concentration and saturation of hemoglobin in brain tissue can fill this gap.

The propagation of NIR light in tissues depends on the spatio-temporal properties of two optical parameters, namely the absorption and the reduced scattering coefficients, according to the diffusion equation (DE). Therefore, absolute quantification of the concentration of hemoglobin chromophores (which are directly derived from the absorption coefficient) requires decoupling the contributions from these two optical coefficients to the optical measurements. This can be fulfilled by time-resolved approaches in the time-domain [36] or in the frequency-domain [37], even though specialized continuous-wave (CW) methods have also been reported [38], [39].

The importance of absolute measurements for *in vivo* interrogation of cerebrovascular health has been shown in animal models [35], [40], [41], [42]. Briefly, Saxena *et al*. performed *in vivo* measurements of absolute hemoglobin concentrations [i.e. oxygenated ([HbO_2_]), deoxygenated ([Hb]), and total ([HbT] = [HbO_2_]+[Hb])] and hemoglobin oxygen saturation (StO_2_ = [HbO_2_]/[HbT]) in mouse brain using a CW approach and reported a strong correlation between brain tumor size and [Hb]. Their longitudinal study that lasted 46 days suggested a hypoxic trend in the tumor region during baseline conditions provided by resting state absolute NIRS measurements [40]. Hallacoglu *et al*. measured the same parameters ([HbO_2_], [Hb], [HbT], and StO_2_) in the frequency-domain in the rat brain during resting state, and hypoxia and hypercapnia challenges. A high correlation of vascular cognitive impairment (VCI) with resting state values but not with relative changes induced by hypoxia or hypercapnia was reported. Absolute values measured in that study also suggested hypoxia-driven angiogenesis on VCI rat model on the basis of intra-individual differences that were measured 10 weeks apart [35]. In other frequency-domain approaches, Kurth *et al*. measured [HbO_2_], [Hb], [HbT] and StO_2_ in piglets, and proposed a threshold for the parameter StO_2_ to prevent cerebral hypoxia–ischemia and associated functional impairment [42]. Finally, Choe *et al*. demonstrated that absolute values of [HbO_2_] and [Hb] can be used to measure hypoxic stress in fetal sheep brain *in utero* and emphasized the potential implications of such measurements for the imaging of the human fetus [41]. Such diagnostic and/or long-term monitoring approaches presented above could only be made possible by quantification of concentration and saturation of hemoglobin in absolute terms.

In humans, absolute NIRS measurements found prominent applications in the longitudinal monitoring of infant brain development [43], [44] as well as in the detection of anomalies, for instance, led by brain injury [45] and respiratory distress [46]. Absolute brain oximetry (namely, measurement of StO_2_ in absolute terms) was predicted to become a way for clinicians to more quickly and noninvasively identify infants and children with altered levels of cerebral and/or somatic tissue oxygenation [47].

**Figure 1:**
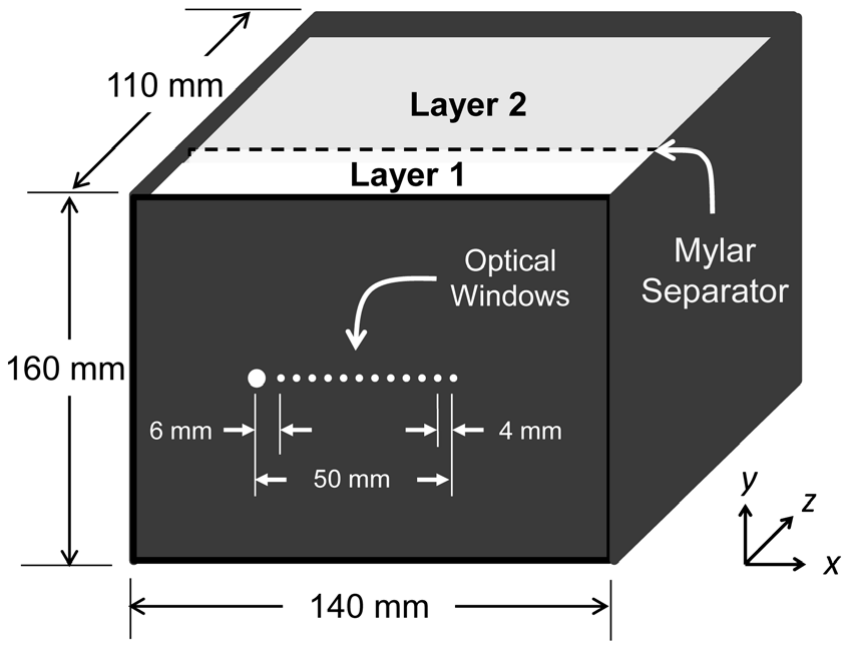
Illustration of the two-layered liquid phantom (Del Bianco *et al*. 2004), where larger and smaller optical windows denote detector and source fiber positions, respectively [**81**].

Despite the success in animal and infant human studies, translation of such methods to adult human brain remains a challenge. Relatively large extracerebral scalp-cortex distance in adult humans (10–23 mm) [48], [49], [50] in comparison to rats (∼1 mm) [35], piglets (∼4 mm) [51], or infants (5–11 mm) [52] creates a problem for traditional light propagation models (i.e. homogenous models) to analyze the NIRS data. While the assumption of tissue homogeneity in the probed volume may be adequate for small animal or infant human brain imaging [53], and may yield robust and reproducible measurements in the adult human brain [37], dependence of the optical coefficients to source detector separations have been reported in several studies [37], [53], [54], [55], [56], [57], indicating a non-negligible depth dependence of the tissue composition. To overcome the limitation of homogenous models, two-layered models have been developed in both the time domain [58] and the frequency domain [59], with experimental validations on tissue-mimicking layered phantoms [60], [61], [62], [63], [64], [65]. *In vivo* application of these approaches to noninvasive brain measurements of absolute optical coefficients have also been investigated in the frequency domain [41], [54] and the time domain [56], and differences with respect to homogenous assumption have been measured in experimental [41] and simulation based [56] data. Briefly, Gagnon *et al*. showed that the homogeneous model underestimated the absolute hemoglobin concentrations in the brain by about 30% (Fig. 1 in their paper) [56]. Choe *et al*. measured absolute changes in StO_2_ during hypoxia (i.e. baseline minus hypoxic state) using homogenous and two-layered models and reported that homogenous model underestimated this quantity by up to 90% (true values were determined using a hemoximeter) [41]. The advantages of using two-layer models over homogenous models in brain imaging have been demonstrated through the works discussed above; however widespread adoption of such approaches has not occurred due to limitations. These limitation include requirement of complementary MRI measurements [54], [56] or invasive means [41] to measure top layer thickness (cortex depth) and inability to measure first and second layer optical properties simultaneously.

In this article, we report absolute measurements of the optical coefficients of two-layered media that expand on our previous work on multi-distance absolute measurements on tissue-like phantoms [66], animal brain [35], [51], and human brain [37]. We introduce an inversion procedure based on the solution of the frequency-domain diffusion equation for a two-layered medium to translate multi-distance optical data into measurements of the absolute values of the optical coefficients in both layers, and of the top layer thickness. When applied to optical measurements on the human head, our method yields hemoglobin concentrations and saturation in the extracerebral tissue (layer 1) and in the cerebral tissue (layer 2), as well as the cortical depth (scalp-cortex distance). Our method, which is based on our own implementation of Levenberg-Marquardt inversion procedure, has been tested by: 1) inverting simulated data generated by two-layered Monte Carlo simulations; 2) inverting experimental data collected on two-layered liquid phantoms; and 3) inverting experimental data collected on the forehead of human subjects *in vivo*. To the best of our knowledge, this is the first report of concurrent measurements of hemoglobin concentration in extracerebral and cerebral tissues combined with a measure of cortical depth using standalone NIRS.

## Methods

### Photon Migration in Turbid Media

Starting from the radiative transfer equation (RTE) in transport theory, which describes light propagation in random media, photon migration in highly scattering media (such as biological tissues) can be approximated by the diffusion equation (DE). Letting µ*_a_*(**r**) and **µ**′*_s_*(**r**) represent the absorption and the reduced scattering coefficients, respectively, and *D*(**r**) = 1/(3 **µ**′*_s_*) the diffusion coefficient in a medium as a function of position vector **r**, the frequency-domain DE for the photon fluence rate 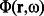 due to an intensity modulated point source, is given by [67]:
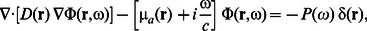
(1)where *c* is the speed of light in the media, 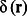 is the Dirac delta function that represents a point-like photon source, and *P* is the frequency-dependent source power (photons/sec). Equation (1) reduces to the continuous wave (CW) diffusion equation by setting the angular modulation frequency (ω) to zero. Analytical solutions of the diffusion equation have been developed and reported in the literature in the time domain [58], [59], [68], [69], [70], frequency domain [59], [67], [68], and CW [69], [71] for homogenous [67], two-layered [58], [59], [69], [70] and N-layered [68] media. Other solutions of the DE for regularly bounded geometries have also been reported [72]. In this work, we have used solutions for both homogenous media (as given in Fishkin and Gratton, 1993) [67] and two-layered media (as given in Kienle *et al*., 1998 [59] for planar geometry, and Liemert and Kienle, 2010 [73] for cylindrical geometry), therefore we shall briefly summarize these solutions in the subsections below.

#### Diffusion model for homogenous media

For a source term described by: 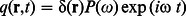 in the frequency-domain (FD), where **r** and *t* are the spatial and temporal variables, respectively, the complex fluence rate at the point of observation in an unbounded (i.e. infinite) homogeneous medium is given by [67]:
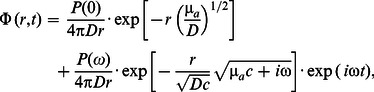
(2)where *D* = 1/(3 **µ**′*_s_*) is the diffusion coefficient, and *r* is the distance between the point source and the point of observation. Note that the second term of Eq. (2) is the solution of Eq. (1) for a homogeneous unbounded medium. Three components of the fluence rate can be extracted from Eq. (2), namely the steady-state, direct current (DC or CW) term, the alternating current (AC) amplitude, and the phase (PH) of the oscillatory term, given by:



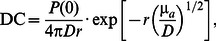
(3)

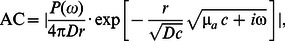
(4)

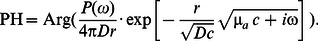
(5)


In the infinite geometry, the following expressions are linearly dependent on *r* in the following form [74]:
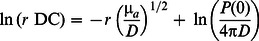
(6)

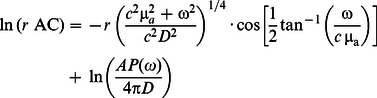
(7)

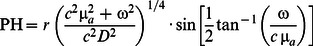
(8)


This linear dependence leads to an easy inversion procedure to evaluate µ*_a_* and **µ**′*_s_* using, for instance, the slopes of the straight lines of ln(*r* AC) and phase (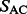 and 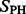, respectively) as functions of *r* [67], [74]:
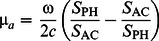
(9)

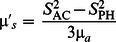
(10)


#### Diffusion model for two-layered media

We have implemented two separate solutions of the DE for two-layered media: First, the solution using the Fourier transform formalism for the planar geometry (i.e. semi-infinite regime), given in [59], and second, the improved solution, which was developed in the cylindrical geometry, given in [73]. We used the latter solution [73] for the analysis presented in this article, because we found it to be computationally faster and more robust at large source-detector separations (*r*>30 mm) and high reduced scattering coefficients (**µ**′*_s_* >1 mm^−1^). We provide a brief description of this solution in the following.

For a point source that is incident onto the center of a layered cylindrical medium, the general solution of the two-layered DE in cylindrical coordinates 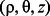 is given by [73]:
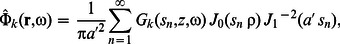
(11)where 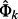 is the photon fluence in the *k* ^th^ layer of the medium (i.e. *k = *1 or 2 for a two-layered medium), *s_n_* are the positive roots of the 0-order Bessel function of first kind divided by *a′ = a*+*z_b_*, (where *a* is the radius of the cylinder), *J_m_* is the Bessel function of first kind and order *m*, *z_b_* = 2*D*_1_ (1+*R*_eff_)/(1−*R*_eff_), and *R*_eff_ is the fraction of photons that are internally diffusely reflected at the cylinder boundary based on [75]. Here, *G*_1_ is defined in the following form:
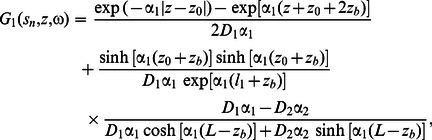
(12)where 
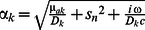
, 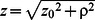 is the distance between the isotropic point source and the point of observation (i.e. location of the detector), and z0 = 1/µ′s1. For the calculation of the diffusely reflected intensity (*R*), we have considered two expressions: the first, which uses only the flux at the boundary according to Fick’s Law [75]:
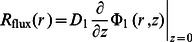
(13)and the second, which uses a combination of fluence rate and flux, given by [76]:
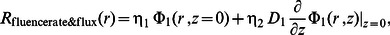
(14)where r represents the source-detector separation, and coefficients η1 and η2 are chosen based on the refractive index mismatch between first layer and surrounding medium [76]. We have used Eq. (13) (i.e. Fick’s law) for the calculation of *R*(*r*) throughout this study, as it showed better agreement with Monte Carlo simulations in this work. In the frequency domain, AC and PH terms are given by:



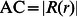
(15)

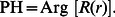
(16)


The DC two-layered solution is obtained as a particular case of the FD solution when ω = 0. In this case, the imaginary part of the complex expression for reflectance reduces to zero. In the two-layered case (in comparison to the homogenous case) the inverse problem becomes much more complicated due to the increased number of unknowns. These unknowns are the absorption and reduced scattering coefficients of layer 1 (µ*_a_*_1_, **µ**′*_s_*_1_) and layer 2 (µ*_a_*_2_, **µ**′*_s_*_2_), the thickness of layer 1 (*L*), and a multiplicative AC factor (AF) and an additive phase term introduced by the measurement apparatus. The phase term can be neglected by considering the differences at different source-detector separations [for instance, PH(*r*_2_)–PH(*r*_1_)]. The solution of this problem requires nonlinear fitting procedures on the measured quantities (PH and either AC or DC as a function of *r*).

### Inversion Procedure

To solve the inverse problem, we implemented a Levenberg-Marquard optimization routine in Matlab (Mathworks Inc, Natick, MA) based on [77] of the form:
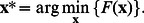
(17)


Here, x* is the result of the optimization, i.e. the best estimates of the unknown parameter vector **x** = [µ*_a_*_1_, **µ**′*_s_*_1_, *L*, µ*_a_*_2_, **µ**′*_s_*_2,_ AF ]*^T^*, which minimizes the cost function:
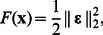
(18)where, **ε** is the error vector composed of the difference between theoretical values and experimental (or simulated) values as:



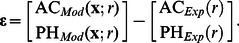
(19)Here, AC*_Mod_* and PH*_Mod_* are the theoretical values yielded from the two-layered model, AC*_Exp_* and PH*_Exp_* are the experimental values either from Monte Carlo simulations or measurements, and 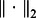 represents the Euclidean norm. In order to employ the Levenberg-Marquardt algorithm, the calculation of the Jacobian matrix **J** of the error vector with respect to the parameter vector is required:
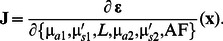
(20)


The solution is then obtained by updating **x** at each iteration as: 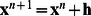, where **h** is obtained by solving the following linear system:
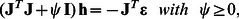
(21)where *ψ* is a damping parameter affecting the size and direction of **h** and found via an appropriate line search algorithm [77]. Once the optimal fit parameters were determined, parameter statistics were computed for the converged solution using weight values equal to the mean square measurement error (namely, errors on AC and PH). The asymptotic standard parameter errors were computed by considering: 
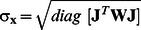
, where 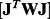 is the covariance matrix, **J** is the Jacobian matrix, and **W** is the weighting matrix, which is constructed as a diagonal matrix with elements representing the inverse square of the measurement errors [78]. Here, *diag* 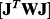 represents a vector constructed with the diagonal elements of the covariance matrix. We considered the measurement errors to be 0.1° and 2% on each PH and AC datum, respectively, which are typical error values of the measurement apparatus used in this work [79].

### Monte Carlo Simulations

Monte Carlo (MC) simulations were performed based on our previous work [80] for the validation of the forward model and testing of the inversion procedure. Briefly, each injected photon was “followed” in its path through the medium until it was either detected, or lost through the medium’s boundary. Moreover the photon was abandoned if its total pathlength (travelled in the medium) exceeded a fixed threshold (chosen differently for each source-detector distance). This corresponded to about three-four decades of the temporal point spread function (Green’s function in time domain). We note that the “late” photons, even if detected, would not contribute to the calculated parameters (DC, AC, and PH). The simulations were run until 10000 photons were detected in each receiver. This number guarantees an accuracy in the DC estimated values of 1% for non-absorbing media (as we chose for our simulations). Scaling relationships based on the microscopic Beer-Lambert law (mbll) were applied in order to scale the results to an arbitrary value of the absorption coefficient. Ten independent simulations were run in order to estimate the errors in the DC, AC and PH.

Simulations were run with refractive index pairs of 1 and 1.4 (for air and two-layered scattering media, respectively), and 1.56 and 1.33 [for surrounding medium (plastic scattering cell) and two-layered medium used in the phantom experiments (Intralipid 20%, Fresenius Kabi, Germany)], according to Mie theory with anisotropy coefficient *g* = 5.15×10^−8^ and a Henyey-Greenstein phase function with *g* = 0.8.

### Experimental Approaches

#### Near infrared instrumentation

All experiments were carried out using a commercial frequency-domain tissue oximeter (OxiplexTS, ISS Inc, Champaign, IL). Two laser diode light sources (emitting at wavelengths of 690 and 830 nm) were intensity modulated at a frequency of 110 MHz and their emitted light delivered to the medium of interest by means of optical fibers. The optical power at the end of the illumination optical fibers was ∼2 mW. On the detection end, another optical fiber was used to deliver the light to a photomultiplier tube, whose gain function was modulated at a slightly different frequency (110 MHz+5 kHz) than that of the intensity modulation of the light sources, giving rise to a beating signal of 5 kHz, which was sent to the computer for analysis. The built in Fast-Fourier-Transform algorithm computed the phase (relative to a reference signal) (PH), the average intensity (DC), and the amplitude of the intensity oscillations (AC) of the detected light. Further details of such frequency-domain system can be found in [79].

#### Two-layered phantom experiments

We used a two-layered liquid phantom [81], where the layers were separated by means of a thin (23 µm) Mylar membrane. Effects of Mylar to diffuse reflectance measurements in the near-infrared region have been shown to be negligible [60]. An illustration of the phantom that was used in this work is provided in Figure 1. The phantom featured thirteen transparent optical windows at the center of its façade including one detection window (3 mm diameter) and twelve illumination windows (1 mm diameter each). Separation of the windows ranged from 6 to 50 mm. The overall dimensions of the phantom to ensure the validity of semi-infinite boundary conditions for layer 2 for a given thicknesses of layer 1 (in *z*-direction) were determined through Monte Carlo simulations. These dimensions are presented in Figure 1. The two layers were filled with controlled suspensions of deionized water, Intralipid 20% (Manufacturer Fresenius Kabi, Germany) and diluted India Ink (weight fraction of ink in water = 0.0074) for background dilution, scattering and absorption characteristics, respectively. Intralipid is an intravenous fat emulsion that has been widely used as a multiply scattering tissue-like phantom [82], [83], [84], [85], [86]. Scattering and absorption coefficients of the two layers were controlled by changing the amount of Intralipid and ink in each layer. We performed multi-distance frequency-domain diffuse reflectance measurements on twelve different two-layered phantom preparations for three different first layer thicknesses (8, 10, and 15 mm). Apart from 8 mm, such first layer thicknesses match true anatomical values of scalp-cortex distance in the adult human forehead (∼12–16 mm) [50]. Data from the two-layered phantoms were analyzed using the matching model and the six-parameter inversion routine described in the previous sections. Even though the data was collected between 6–50 mm source-detector distances, the range considered for the analysis was 14–50 mm (i.e. starting from the third optical window) to comply with the diffusion conditions.

#### Homogenous phantom experiments

Homogenous phantom measurements were conducted in a large container in order to obtain an effectively infinite geometry arrangement, in which optical detector and source fibers (both 400 µm in diameter) were deeply immersed into the medium (at a depth of ∼60 mm) and parallel to each other. This is a typical experimental arrangement for absolute optical characterization of liquid phantoms using NIRS, where the diffused intensity is generated and detected only within the diffusing medium avoiding any complications due to the boundary conditions. We followed a common experimental protocol [74], where we employed an illumination fiber (400 µm diameter) delivering 830 nm wavelength of light and linearly scanned it away from the collection fiber by means of a programmable mechanical linear stage scanning system (Velmex Inc., Bloomfield, NY, USA) over the source–detector distances of ∼20 to 35 mm. This procedure was repeated twenty times for eight different homogenous phantom preparations. The acquisition was automated and controlled by a program written in LabVIEW software (National Instruments, Austin, TX, USA). By exploiting the linear dependence of µ*_a_* and **µ**′*_s_* on the concentration of ink and Intralipid, respectively, we obtained: 1) intrinsic optical coefficients of pure Intralipid and ink; and 2) linear expressions that provided an independent means to characterize each medium that was used for the two-layered measurements.

#### Human subject experiments

**Figure 2:**
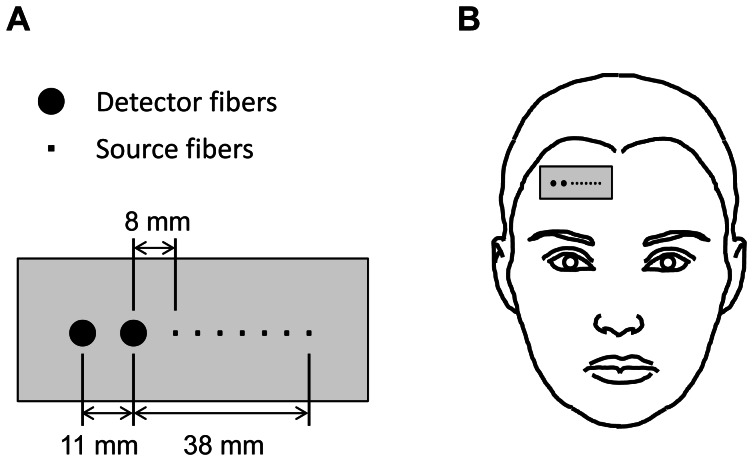
Schematic diagram of the experimental setup for the human subject measurements. A) The polystyrene optical probe used in the *in vivo* measurements, and (B) its positioning on the subject’s forehead during measurements. Larger and smaller circles in (A) denote detector and source fibers positions, respectively.



Participants were 3 healthy subjects (all males, mean±standard deviation age of 29±2 years). The protocol for the human subject measurements was approved by the Tufts University Institutional Review Board and written informed consent was obtained from all subjects prior to the measurements. We used an approach based on a preliminary calibration of the optical probe on a known phantom as described in [37]. The optical probe was made of polyurethane silicon and featured two detector optical fiber bundles (3 mm in core diameter) separated by 11 mm, and seven pairs of illumination optical fibers (0.4 mm in core diameter) that guided light at 690 and 830 nm, located at distances in the range 8–48 mm from the detector fiber bundles. Figure 2 illustrates the probe layout and placement on the subject’s forehead. Source fibers were located 8 to 38 mm from the first detector bundle and 18 to 48 mm from the second detector bundle in 5 mm increments. The optical probe was placed on the left side of the subjects’ forehead and held in place using a commercial sports band to exert light pressure for comfort, while guaranteeing good contact between the optical fibers and the subjects’ scalp. For the analysis, recordings from the two detector bundles were combined, providing an effective source-detector range of 8 to 48 mm. Here, the range considered for the analysis was 13–48 mm to comply with the diffusion conditions. Under the assumption that oxy-hemoglobin (HbO_2_), deoxy-hemoglobin (Hb) and water are the major absorbers in the probed tissue volume at the two wavelengths considered, we calculated tissue concentrations of HbO_2_ and Hb in each layer using the following expressions [37]:
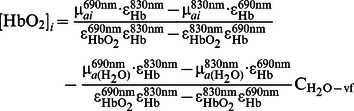
(25)

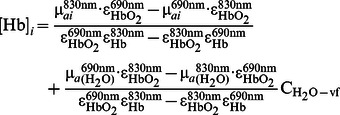
(26)where ε_Hb_ and ε_HbO2_ are the molar extinction coefficients of Hb and HbO_2_ [87], respectively, µ*_a_*_ (H2O)_ is the absorption coefficient of water [88], and C_H2O-vf_ is the volume fraction of water content in the probed tissue volume for *i* = 1, 2 (i.e. extracerebral and cerebral tissue, respectively). Here, we assume a value of 0.7 (i.e. 70% water content) for C_H2O-vf._ [37], [56], [57] Finally, we calculated the total hemoglobin concentration and hemoglobin saturation in each layer using:

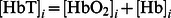
(27)

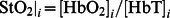
(28)


## Results

### Results of the Monte Carlo Simulations

**Figure 3:**
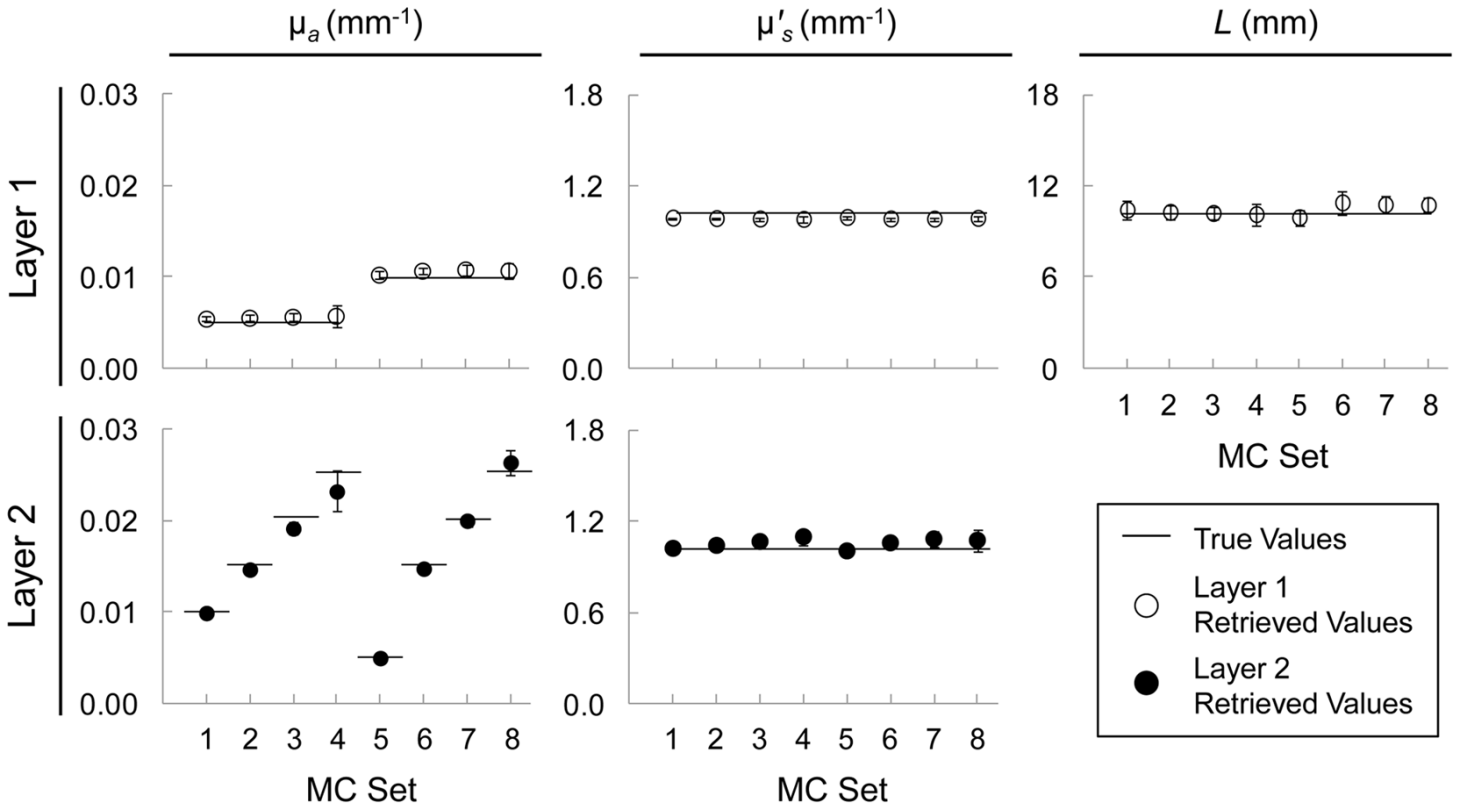
Six-parameter fitting procedure results on eight sets of two-layered Monte Carlo (MC) simulations reported in terms of MC set. *True values* represent values used for µ*_a_* (column 1), **µ**′*_s_* (column 2), and first layer thickness (column 3) in each MC simulation. Retrieved values for Layer 1 (row 1) and Layer 2 (row 2) are represented by open and filled circles, respectively. The error bars represent uncertainties determined by the fitting procedure.

Recovered values from all eight Monte Carlo (MC) data sets were in good agreement with the actual values for all five parameters (µ*_a_*_1_, **µ**′*_s_*_1_, *L*, µ*_a_*_2_, **µ**′*_s_*_2_). We report these results in Figure 3 in terms of MC data set (from 1 through 8), where the error bars represent uncertainties associated with the fitting procedure. All five parameters for most MC sets were recovered within 10% of the actual values. In two of the simulations (namely, MC sets: 3 and 4), where the absorption coefficient of layer 1 (µ*_a_*_1_ = 0.005 mm^−1^) was significantly different than that of layer 2 (µ*_a_*_2_ = 0.02 mm^−1^ for No. 3; and µ*_a_*_2_ = 0.025 mm^−1^ for No. 4), µ*_a_*_1_ values were recovered with slightly larger errors (i.e. 12% for No. 3; and 14% for No. 4). However, deviations from the actual values for all other parameters in these simulations were still <10%. We note that the discrepancy between MC and DE would mostly account for these errors. To investigate this point, we have also carried out separate measurements on synthetic data generated using our diffusion based forward model with additive noise (typically 1% random noise). We found a more accurate recovery of the true values in these data sets, with deviations never exceeding a few percent and always smaller (as it is expected) than those found in the MC data (data not shown).

Our sensitivity analysis showed negligible effects of initial guesses for the parameter values in the inversion procedure. We demonstrate the method’s lack of sensitivity to initial guesses in Table 1, where two sets of initial guesses (i.e. “good” set and “bad” set) and retrieved values on the same representative data set is reported. As demonstrated in the Table 1, deviations between the retrieved and true values were less than 7% in both cases. This is a particularly important result demonstrating the method’s lack of sensitivity to local minima.

**Table 1:** Demonstration of the insensitivity of the inversion procedure to initialization.

	µ*_a_*_1_ (mm^−1^)	µ′*_s_*_1_ (mm^−1^)	*L* (mm)	µ*_a_*_2_ (mm^−1^)	µ′*_s_*_2_ (mm^−1^)
True Values	0.0050	1.00	10.0	0.0100	1.00
**Good Initial Guess**	0.0060	1.20	8.0	0.0120	1.20
Retrieved Values	0.0053	0.99	10.2	0.0098	1.02
*Error on Initial Guess*	*20%*	*20%*	*−20%*	*20%*	*20%*
*Error on Retrieved Values*	*7%*	*−1%*	*2%*	*−2%*	*2%*
**Bad Initial Guess**	0.0100	3.00	1.0	0.0200	2.00
Retrieved Values	0.0054	0.99	10.4	0.0099	1.02
*Error on Initial Guess*	*100%*	*200%*	*−90%*	*100%*	*100%*
*Error on Retrieved Values*	*8%*	*−1%*	*4%*	*−1%*	*2%*

Similar convergence to good and bad initialization is reported.

### Results of the Phantom Measurements

#### Homogeneous phantom results

**Figure 4:**
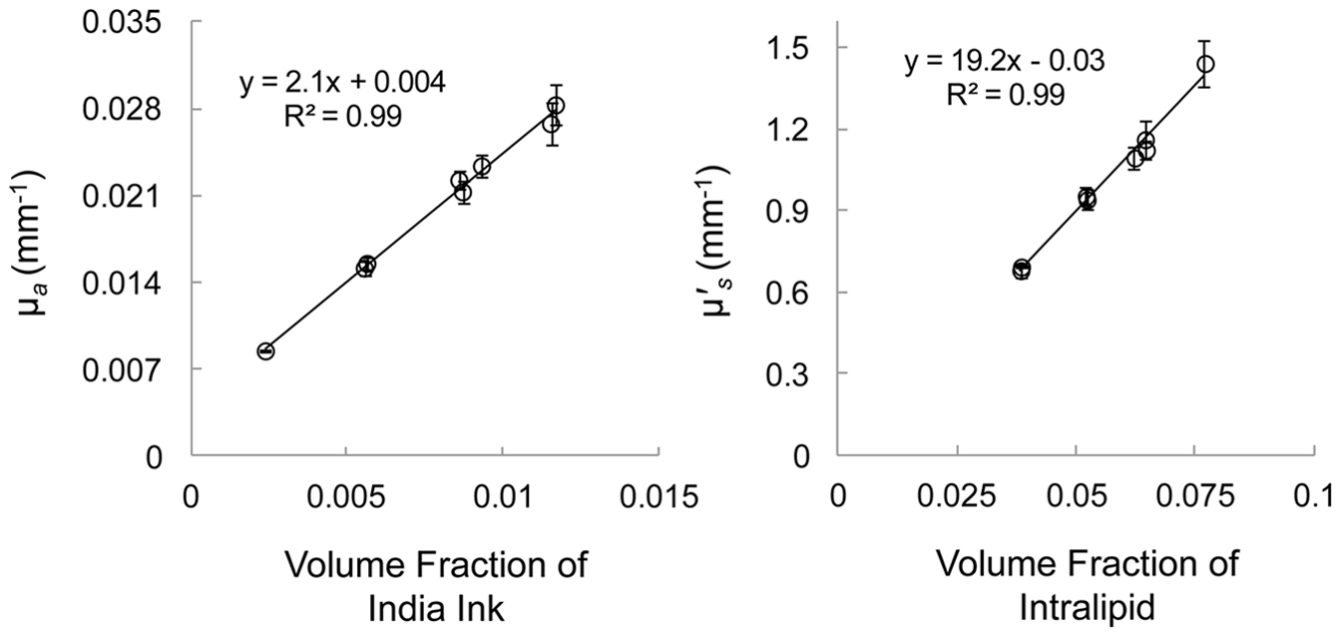
Measurement of µ*_a_* (left panel) and µ′*_s_* (right panel) at 830 nm wavelength on eight homogenous phantoms in the infinite geometry, presented as a function of volume fractions of ink dilution and intralipid, respectively. The symbols and error bars represent mean and standard deviation of 20 independent measurements in each phantom. The slopes of the linear curves were used to characterize the precise quantities used in the two-layered phantom measurements.

In our infinite medium measurements on eight liquid homogenous phantoms, we found a linear dependence of µ*_a_* and **µ**′*_s_* on India ink and Intralipid concentrations, respectively. These results are shown in Figure 4, whereby we report measured µ*_a_* and **µ**′*_s_* at 830 nm as a function of volume fractions of diluted-ink and Intralipid. These are typical results for such experiments in the infinite geometry [74], [89], [90], which in fact confirm that the infinite boundary conditions were met during our measurements. We performed linear regression analysis on the experimental data and computed the slopes of µ*_a_* and **µ**′*_s_* to be 2.1±0.1 (mm^−1^/%diluted-ink) and 19.2±1.2 (mm^−1^/% Intralipid), respectively. We note that these slopes represent the intrinsic µ*_a_* and **µ**′*_s_* per unit concentration of diluted-ink and pure-intralipid, respectively, which were used for the accurate optical characterization of the suspensions used in the two-layered phantom measurements. We evaluated the intrinsic µ*_a_* of pure-ink to be 281.8±13.6 (mm^−1^/% ink). This led to infinite medium measurement uncertainties of ∼6% for both µ*_a_* and **µ**′*_s_*. The *y*-intercepts of diluted-ink and Intralipid regression lines (0.004±0.001 mm^−1^ and *−*0.03±0.04 mm^−1^, respectively), which represent our measured µ*_a_* and **µ**′*_s_* of pure water (Figure 4). Although these values should be equal to the actual µ*_a_* and **µ**′*_s_* of water at 830 nm wavelength (µ*_a_* = ∼0.003 mm^−1^ [88] and **µ**′*_s_* = 0 mm^−1^, respectively), the actual values were within the measurement uncertainties.

#### Two-layered phantom results

In Figure S1 (supplementary material), we report the results of a representative two-layered phantom measurement including the resulting fit (solid lines) on measurements (circles) of AC (left panel) and phase (right panel) as a function of source-detector distance. Dotted lines illustrate the results yielded by the first iteration of the inversion procedure (using the initial guesses). In Figure S2, we present the evolution of the six fitted parameters [µ*_a_*_1_, µ′*_s_*_1_, *L*, µ*_a_*_2_, µ′*_s_*_2,_ AF] for the same phantom considered in Figure S1 during all iterations involved in the fitting procedure, which demonstrate the robustness of the inversion procedure and once again its insensitivity to initial guesses. We are presenting results every other iteration for clarity. Horizontal solid and dashed lines represent the true values, measured independently in the infinite geometry using a homogenous model as described above in the homogenous phantom experiments subsection.

**Figure 5:**
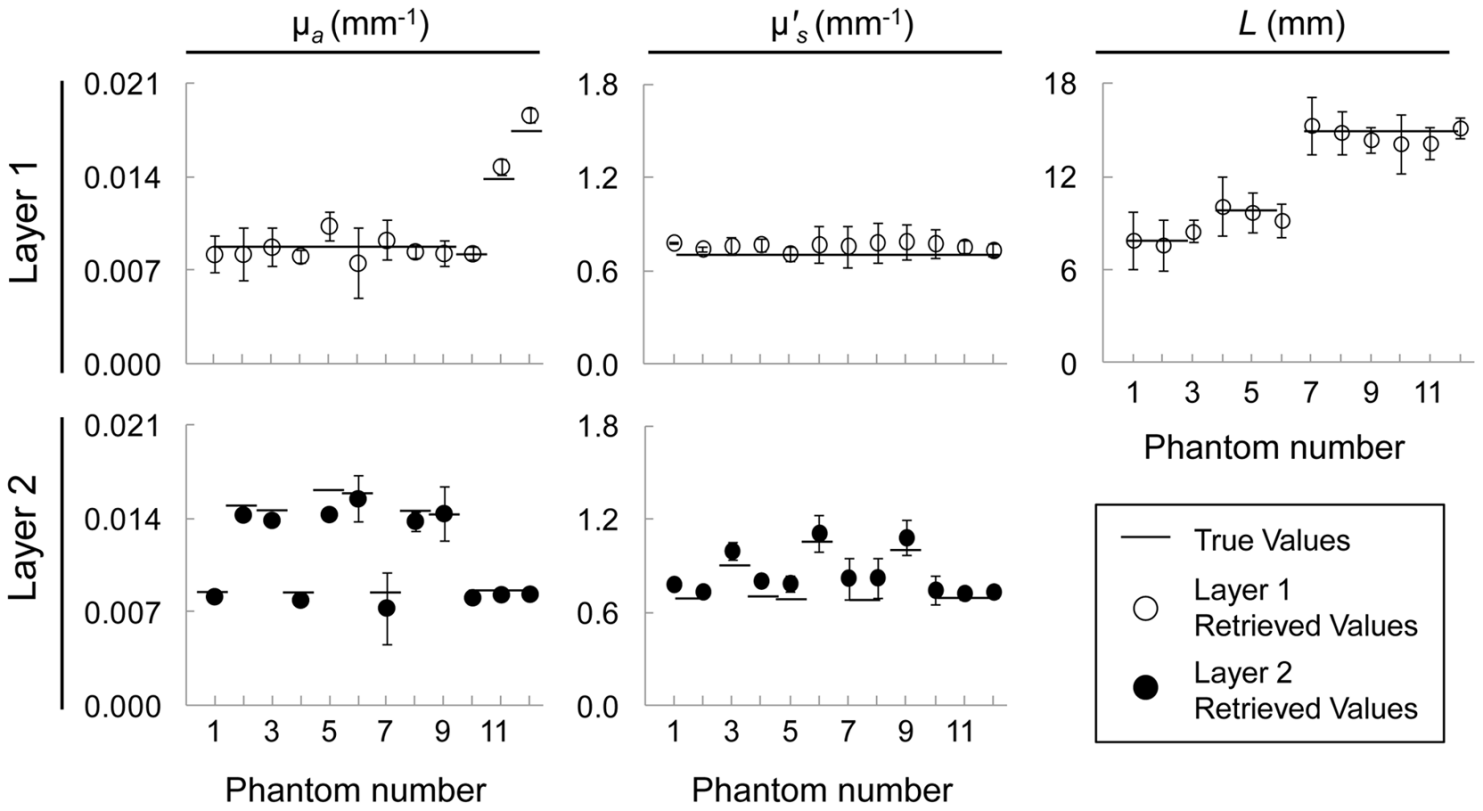
Six-parameter fitting procedure results on twelve two-layered phantoms with a range of optical coefficients and top-layer thicknesses that are representative of the human brain. *True values* represent quantities measured in the infinite medium geometry using a homogenous model. Retrieved values for layer 1 (row 1) and layer 2 (row 2) are represented by open and filled circles, respectively. The error bars represent uncertainties determined by the fitting procedure.

**Figure 6:**
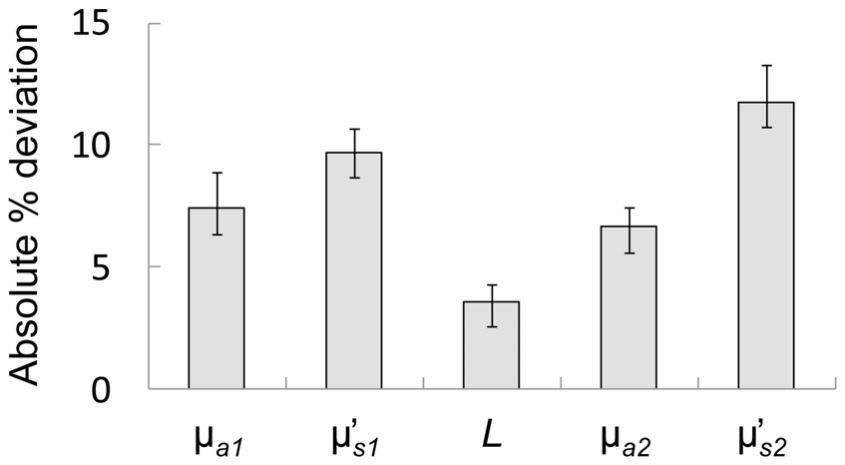
Average of the absolute % errors in the measured parameters with respect to true values across twelve two-layered phantoms. The error bars represent standard errors.

Results of the two-layered phantom measurements for all twelve phantoms are reported in Figure 5, where the error bars represent uncertainties associated with the fitting procedure. Considering the complexity of liquid phantom measurements, we have found excellent agreement between the true values and the retrieved values (i.e. measured from two-layered phantoms and retrieved simultaneously using our two-layered inversion routine). In fact, taking into account all two-layered phantom measurements [i.e. 12 phantoms ×5 key parameters (µ*_a_*_1_, µ′*_s_*_1_, *L*, µ*_a_*_2_, µ′*_s_*_2,_) = 60 parameters in total] more than 75% of the retrieved parameters were within 10% of the true values, half of which were within 5% of the true values. More specifically, the most accurately retrieved parameters in the two-layered phantom measurements were *L* and µ*_a_*_2_, with ranges of absolute % deviations of 1%–8% and 3%–13%, respectively, from the true values. Subsequently, ranges of absolute % deviations in µ*_a_*_1_, µ′*_s_*_1_, µ′*_s_*_2_ from their true values were 1%–14%, 3%–15%, and 5%–20%, respectively. Therefore, the least accurately retrieved parameter was µ′*_s_*_2_, where in four of the twelve phantoms we observed absolute % deviations within 15%–20% (Nos. 4, 7, 8 and 9) from the true values. The complete summary of the phantom experiments is reported in Table 2, where we also include retrieved values using a semi-infinite homogenous model for comparison. Considering the measurement uncertainties in the true values (∼6%) and in the retrieved values (as shown by the error bars in Fig. 5), as well as mixture calibration errors during the liquid phantom preparation (not quantified), the two-layered approach employed here was able to retrieve all parameters with good accuracy. We present the absolute % deviation in the measured parameters averaged across 12 phantoms in Figure 6, where the error bars represent standard errors of the % deviations. We underscore the fact that clinically most relevant parameters, *L* and µ*_a_*_2_, which are representative of scalp-cortex distance and cerebral absorption coefficient, respectively, were recovered with high accuracy (absolute % error <7%).

**Table 2:** Summary of the phantom experiments.

		Two-Layered					Homogenous	
		μ*_a_*_1_ (mm^-1^)	μ*'_s_*_1_ (mm^-1^)	*L* (mm)	μ*_a_*_2_ (mm^-1^)	μ'*_s_*_2_ (mm^-1^)	μ*_a_*(mm^-1^)	μ'*_s_* (mm^-1^)
Phantom 1	True Value	0.009	0.69	8.0	0.009	0.69	0.009	0.69
	Retrieved Value	0.008	0.78	7.8	0.008	0.78	0.008	0.78
	Error	*-7%*	*13%*	*-3%*	*-7%*	*13%*	*-7%*	*13%*
Phantom 2	True Value	0.009	0.69	8.0	0.015	0.68	0.015	0.68
	Retrieved Value	0.009	0.74	8.0	0.014	0.73	0.013	0.70
	Error	*-2%*	*7%*	*-1%*	*-4%*	*7%*	*-13%*	*2%*
Phantom 3	True Value	0.009	0.69	8.0	0.015	0.93	0.015	0.93
	Retrieved Value	0.009	0.76	8.5	0.014	0.99	0.014	0.77
	Error	*0%*	*10%*	*6%*	*-6%*	*7%*	*-9%*	*-17%*
Phantom 4	True Value	0.009	0.69	10.0	0.009	0.69	0.009	0.69
	Retrieved Value	0.008	0.77	10.1	0.008	0.80	0.008	0.78
	Error	*-8%*	*12%*	*1%*	*-10%*	*17%*	*-8%*	*13%*
Phantom 5	True Value	0.009	0.69	10.0	0.017	0.68	0.017	0.68
	Retrieved Value	0.010	0.71	9.7	0.014	0.78	0.013	0.69
	Error	*18%*	*3%*	*-3%*	*-13%*	*15%*	*-19%*	*1%*
Phantom 6	True Value	0.009	0.69	10.0	0.016	1.02	0.016	1.02
	Retrieved Value	0.008	0.77	9.2	0.016	1.11	0.014	0.75
	Error	*-14%*	*11%*	*-8%*	*-5%*	*8%*	*-16%*	*-27%*
Phantom 7	True Value	0.009	0.68	15.0	0.009	0.68	0.009	0.68
	Retrieved Value	0.009	0.76	15.6	0.008	0.80	0.009	0.77
	Error	*5%*	*11%*	*4%*	*-6%*	*17%*	*1%*	*13%*
Phantom 8	True Value	0.009	0.68	15.0	0.015	0.68	0.015	0.68
	Retrieved Value	0.009	0.72	14.7	0.014	0.82	0.010	0.74
	Error	*3%*	*5%*	*-2%*	*-7%*	*21%*	*-32%*	*9%*
Phantom 9	True Value	0.009	0.68	15.0	0.015	0.92	0.015	0.92
	Retrieved Value	0.008	0.79	14.4	0.014	1.08	0.010	0.74
	Error	*-6%*	*15%*	*-4%*	*-3%*	*17%*	*-34%*	*-20%*
Phantom 10	True Value	0.009	0.69	15.0	0.009	0.69	0.009	0.69
	Retrieved Value	0.008	0.77	14.1	0.008	0.74	0.008	0.77
	Error	*-4%*	*12%*	*-6%*	*-8%*	*8%*	*-7%*	*13%*
Phantom 11	True Value	0.013	0.69	15.0	0.009	0.69	0.009	0.69
	Retrieved Value	0.015	0.75	14.2	0.008	0.72	0.011	0.82
	Error	*10%*	*9%*	*-6%*	*-5%*	*5%*	*30%*	*20%*
Phantom 12	True Value	0.017	0.69	15.0	0.009	0.69	0.009	0.69
	Retrieved Value	0.019	0.73	15.2	0.008	0.73	0.014	0.83
	Error	*12%*	*6%*	*1%*	*-5%*	*6%*	*59%*	*21%*

*True Value* represents optical coefficients measured in infinite medium geometry, and the actual thickness of the first layer; *Retrieved Value* represents two-layered phantom measurements retrieved using two-layered or homogenous inversion procedures; and *Error­* represents the error between the true values and the retrieved values.

### Results of the Human Subject Measurements

We report retrieved values for µ*_a_*_1_, µ′*_s_*_1_, *L*, µ*_a_*_2_, µ′*_s_*_2_ for wavelengths 690 nm and 830 nm in Table 3. A first striking result is the reproducibility of *L*, which was measured independently using the two wavelengths (Table 3). The measured range for this parameter across all subjects (11.4–14.5 mm) are within anatomical values reported in the literature for the adult human forehead (∼12–16 mm) [50]. For a comparison with Table 3, in Table 4, we report optical coefficients that were retrieved using a semi-infinite homogenous model. In Tables 5 and 6, we report corresponding hemoglobin values. Other common characteristics across subjects include significantly higher µ*_a_*_2_ values with respect to µ*_a_*_1_ (Table 3), which corresponds to higher total hemoglobin concentrations in the cerebral tissues (82±14 µM) in comparison to superficial tissues (26±7 µM) (Table 6). We also found slightly higher hemoglobin saturation in cerebral tissues (62%±10%) in comparison to superficial tissues (56%±6%); however this difference was not significant (Table 6).

**Table 3:** Retrieved optical coefficients and first layer thickness on the forehead of three human subjects using the two-layered model.

	µ*_a_*_1_ (mm^−1^)		µ′*_s_*_1_ (mm^−1^)		*L* (mm)		µ*_a_*_2_ (mm^−1^)		µ′*_s_*_2_ (mm^−1^)	
Subject	690 nm	830 nm	690 nm	830 nm	690 nm	830 nm	690 nm	830 nm	690 nm	830 nm
1	0.007	0.006	1.2	1.1	11.8	11.4	0.019	0.016	0.2	0.2
2	0.009	0.009	1.3	1.2	13.0	12.3	0.020	0.020	0.4	0.3
3	0.008	0.008	1.3	1.1	14.5	14.0	0.022	0.023	0.4	0.2
*mean*	*0.008*	*0.008*	*1.3*	*1.1*	*13.1*	*12.6*	*0.020*	*0.020*	*0.3*	*0.2*
*stdev*	*0.001*	*0.002*	*0.0*	*0.0*	*1.3*	*1.3*	*0.002*	*0.003*	*0.1*	*0.1*

Group mean and standard deviation (stdev) is reported in the bottom two rows.

**Table 4:** Retrieved optical coefficients on the forehead of three human subjects using a homogenous model as a comparison with Table 3.

	µ*_a_* (mm^−1^)		µ′*_s_* (mm^−1^)	
Subject	690 nm	830 nm	690 nm	830 nm
1	0.013	0.012	0.4	0.3
2	0.011	0.013	1.0	0.7
3	0.010	0.012	0.8	0.6
*mean*	*0.011*	*0.012*	*0.7*	*0.5*
*stdev*	*0.001*	*0.000*	*0.3*	*0.2*

Group mean and standard deviations (stdev) are reported in the bottom two rows.

**Table 5:** Retrieved concentrations of oxy-hemoglobin ([HbO_2_]) and deoxy-hemoglobin ([Hb]) on the forehead of three human subjects using two-layered (indicated by ‘Superficial’ and ‘Cerebral’) and homogeneous models.

	[HbO] (µM)			[Hb] (µM)		
Subject	Superficial	Cerebral	Homogeneous	Superficial	Cerebral	Homogeneous
1	8.0	38.0	25.6	9.9	30.3	21.1
2	19.8	51.8	33.8	12.4	31.2	14.3
3	17.1	63.7	31.8	10.5	31.9	14.5
*mean*	*15.0*	*51.2*	*30.4*	*10.9*	*31.1*	*16.6*
*stdev*	*6.2*	*12.9*	*4.3*	*1.3*	*0.8*	*3.9*

Group mean and standard deviations (stdev) are reported in the bottom two rows.

**Table 6:** Retrieved total hemoglobin concentration ([HbT]) and hemoglobin oxygen saturation (StO_2_) on the forehead of three human subjects using two-layered (indicated by ‘Superficial’ and ‘Cerebral’) and homogeneous models.

	[HbT] (µM)			StO_2_ (%)		
Subject	Superficial	Cerebral	Homogeneous	Superficial	Cerebral	Homogeneous
1	17.9	68.3	46.7	44.8	55.6	54.7
2	32.2	83.0	48.1	61.5	62.4	69.2
3	27.6	95.6	46.3	61.9	66.6	68.7
*mean*	*25.9*	*82.3*	*47.0*	*56.0*	*61.6*	*64.2*
*stdev*	*7.3*	*13.6*	*0.9*	*9.8*	*5.6*	*8.2*

Group mean and standard deviations (stdev) are reported in the bottom two rows.

## Discussion

### Monte Carlo Simulations

Monte Carlo methods offer a flexible and accurate approach toward simulating photon transport in turbid media. In this work, MC simulations allowed us to verify the validity of our implementation of the analytical solution of the DE for two-layered media. The optical coefficients and top layer thickness in all MC sets were recovered with good accuracy, with deviations in the order of a few percent in most cases, which are typical uncertainties found also in other studies [59], [63]. To test the performance of our inversion routine, we have also carried out separate measurements on synthetic data generated using our diffusion based forward model with additive noise (typically 1% random noise). We found accurate recovery of the true values in these data sets, with deviations never exceeding a few percent and always smaller (as it is expected) than those found in the MC data (data not shown).

### Phantom Measurements

The two-layered phantom experiments presented here were of key importance in the validation of our approach, which featured an unconstrained inversion procedure of six unknown parameters with no prior knowledge. Previous experiments on two-layered phantoms, featuring two-layered models have been reported for the representation of extracerebral tissue overlaying cerebral tissue [61] (top layer thickness typically more than ∼10) and skin tissue overlaying fat/muscle tissue [60], [62], [63], [64], [65] (top layer thickness typically less than ∼6 mm). In just two of these studies optical coefficients of the two layers and the top layer thicknesses were measured simultaneously [60], [63]. In the frequency domain, Alexandrakis *et al*. used a simplex search algorithm and recovered the second layer optical coefficients with good accuracy (absolute % deviations ∼10%) in almost all phantoms [63]. However their measurement accuracy was lower for the first layer parameters. In the time domain, Martelli *et al*. used a Levenberg-Marquardt routine and reported excellent recovery of the first layer optical coefficients and second layer absorption coefficient (with absolute % deviations of less than 5%) [60]. For the first layer thickness and the second layer scattering coefficients their accuracy was lower (absolute % deviations of 11% and 33%, respectively). In the current study, using a frequency domain acquisition system and a Levenberg-Marquardt inversion routine we were able to measure all parameters with relatively high accuracy (with absolute % deviations of less than 12% for all parameters) (as seen in Figure 6). To our knowledge, such high accuracy measurements on two-layered phantoms in the frequency domain are unprecedented. Furthermore, the experimental tests (where the reference true values were obtained with diffusion theory in an infinite geometry and with source-detector separations >20 mm, thus under conditions well within the limits of applicability of diffusion theory) indicate the accuracy of our proposed diffusion-based method for the optical characterization of two-layered media. In comparison with the two-layered analysis, homogenous analysis performed: *i*) reasonably well in phantoms with 8 mm first layer thicknesses (Nos. 2 and 3); *ii*) worse in phantoms with 10 mm first layer thicknesses (Nos. 5 and 6); and *iii*) much worse in phantoms with 15 mm first layer thicknesses (Nos. 8,9,11 and 12) in retrieving the second layer absorption coefficient (µ*_a_*_2_) (Table 2). We note that such results are indicative of the adequacy of homogeneous analysis for thin first layer thicknesses (here, <8 mm) in the source-detector distances that were considered (14–50 mm). Furthermore, the same comparison in the homogenous phantoms (Nos. 1, 4, 7 and 10) highlights the robustness of the two-layered analysis in homogeneous media.

### Human Subject Measurements

Although we have not performed independent validation measurements (for example an MRI measurement of scalp-cortex distance), two arguments can be made in support of the reliability of the results of our method. The first argument is that the reduced scattering coefficient measured at 690 nm was larger than that of 830 nm for both layers and for all subjects, following the expected wavelength dependence relationship [91]. The second argument is the consistency of independently measured scalp-cortex distance at the two wavelengths used in all subjects (Table 3) and that all measured values are within anatomical ranges [50]. We emphasize the importance of such results given that the inversion procedure implemented by us was unconstrained, allowing the measured parameters to take any possible value during the inversion procedure. This point is demonstrated in Figure S2, where the first layer µ*_a_* (µ*_a_*_1_) varies from as high as 0.045 mm^−1^ (around 20^th^ iteration) to as low as ∼*−*0.005 mm^−1^ (around 50^th^ iteration) in the course of the inversion procedure prior to converging to the correct value.

Concentration and saturation of hemoglobin measurements in all three subjects were within previously reported values in animal [35], [51] and human [37], [43], [56] brain. The first and the second layer measurement of some of these parameters (Tables 5 and 6) were significantly different. Such differences have also been observed by other researchers [54], [56]. The advantage of our approach over these earlier studies was the derivation of the hemoglobin parameters and the scalp-cortex distances without any *a priori* knowledge (these studies used MRI to measure the scalp-cortex distance). Interestingly, the total hemoglobin concentration measured in young subjects using a homogenous model in our earlier study (mean±standard deviation: 52±13 µM) [37] compares well with the current study (mean±standard deviation: 47±1 µM) when a homogenous model is used (Table 6). Moreover, these values are in between the first (30±7 µM) and the second layer (82±13 µM) values measured in the current study (Table 6). This is an indication that homogenous assumption was influenced by the extracerebral tissue physiology. Cerebral saturation in the earlier study (mean±standard deviation: 58%±13% ) [37] compares well with both the first (56%±10%) and the second (62%±6%) layer values, as well as the homogenous values (64%±8%) reported here.

Intersubject variability in baseline human head hemoglobin concentrations has been reported in several studies including those that used homogenous [37], [55] or two layered [54], [56] models. Although we have studied only a small group of subjects, in this study we have observed a similar intersubject variability in the baseline concentrations of hemoglobin, both in the extracerebral and cerebral tissue layers (Table 3). These results emphasize the fact that individualized characterization of the baseline optical coefficients of the human head is important and should be a part of routine practice in NIRS and fNIRS studies.

We point out that second layer reduced scattering coefficients measured in the subjects (0.3±0.1 mm^−1^ and 0.2±0.1 mm^−1^ at 690 nm and 830 nm, respectively) appear to be low, even though this result is consistent with some reported values in the literature (such as 0.3 mm^−1^ and 0.5 mm^−1^ as reported in Gagnon *et al*., (Fig. 3A) [56]). Low µ′*_s_*_2_ values such as these may have a physiological origin in that they may be representative of contributions from the clear cerebrospinal fluid (CSF) (featuring low scattering) to the layer 2 measurements. In this case, absorption coefficient in layer 2 (µ*_a_*_2_) would also be underestimated due to the CSF (µ*_a_*_(CSF)_ <0.005 mm^−1^), which would further enhance the differences in hemoglobin concentrations between the two layers. Another possibility is that this parameter may have a low level of information content inherent to the two-layered diffuse reflectance measurements, which is an interpretation also shared by a similar study in the time domain [60]. In fact, µ′*_s_*_2_ was the parameter that we were least sensitive to in our phantom experiments as seen in the absolute % deviation plot (Figure 6).

### Conclusions

We have presented a multi-distance frequency-domain NIRS approach that relies on a two-layered solution of the diffusion equation and an accompanying six-parameter inversion routine to simultaneously measure the absolute optical coefficients of two-layered turbid media. When applied to non-invasive NIRS measurements on the human head, our approach yields the concentration and oxygen saturation of hemoglobin in the extracerebral (layer 1) and cerebral (layer 2) tissue layers, as well as the scalp-cortex distance. This is the first frequency-domain study that presents high accuracy measurement of these parameters simultaneously using standalone NIRS. We have reported results of Monte Carlo (MC) simulations and two-layered phantom measurements. We have also reported measurements in human subjects to explore the *in vivo* applicability of our approach and found physiologically reasonable values for all measured parameters. Looking forward, further studies on a larger subject group would be required to gain confidence in the reproducibility of the absolute values in human subjects. Other potential *in vivo* experiments include studies on animal models featuring measurements under controlled physiological challenges, which would allow for a direct validation of our approach *in vivo*. Moreover, utilizing numerical forward models for a realistic human head geometry would be highly relevant (for instance, using NIRFAST software) [92] to test the performance of our approach in more realistic geometries.

This study has tackled one of the major issues faced by non-invasive optical measurements of the brain, namely the effect of superficial, extracerebral tissue layers. The capability of achieving depth discrimination is highly significant in functional brain studies and in absolute brain oximetry. Our results represent a step toward the goals of performing depth resolved NIRS and absolute measurements of the concentration and saturation of hemoglobin in cortical tissue.

## Supporting Information

Figure S1 **A representative case illustrating the final fit (solid lines) on measurements (circles) of AC attenuation (left panel) and phase shift (right panel) as a function of source-detector distance from the two-layer phantom.** Dotted lines illustrate the results yielded by the first iteration of the inversion procedure (using the initial guesses). Final fit was obtained at the 102^nd^ iteration.
(TIF)

Figure S2 **A representative case illustrating the evolution of the six parameters (µ** ***_a_***_**1**_ **, µ′** ***_s_***_**1**_ **,** ***L*** **, µ** ***_a2_*** **, µ′** ***_s_***_**2**_ **and AF) during the fitting procedure on two-layered phantom data, demonstrating the robustness of the inversion procedure and its insensitivity to initial guesses.** Solid and dashed lines represent true values, measured independently in the infinite geometry.
(TIF)
