# Supplementary figures and images for: Optical Characterization of Two-Layered Turbid Media for Non-Invasive, Absolute Oximetry in Cerebral and Extracerebral Tissue
Source: PLoS One. 2013 May 21;8(5):e64095. doi: 10.1371/journal.pone.0064095 (PMC3660388; doi:10.1371/journal.pone.0064095)

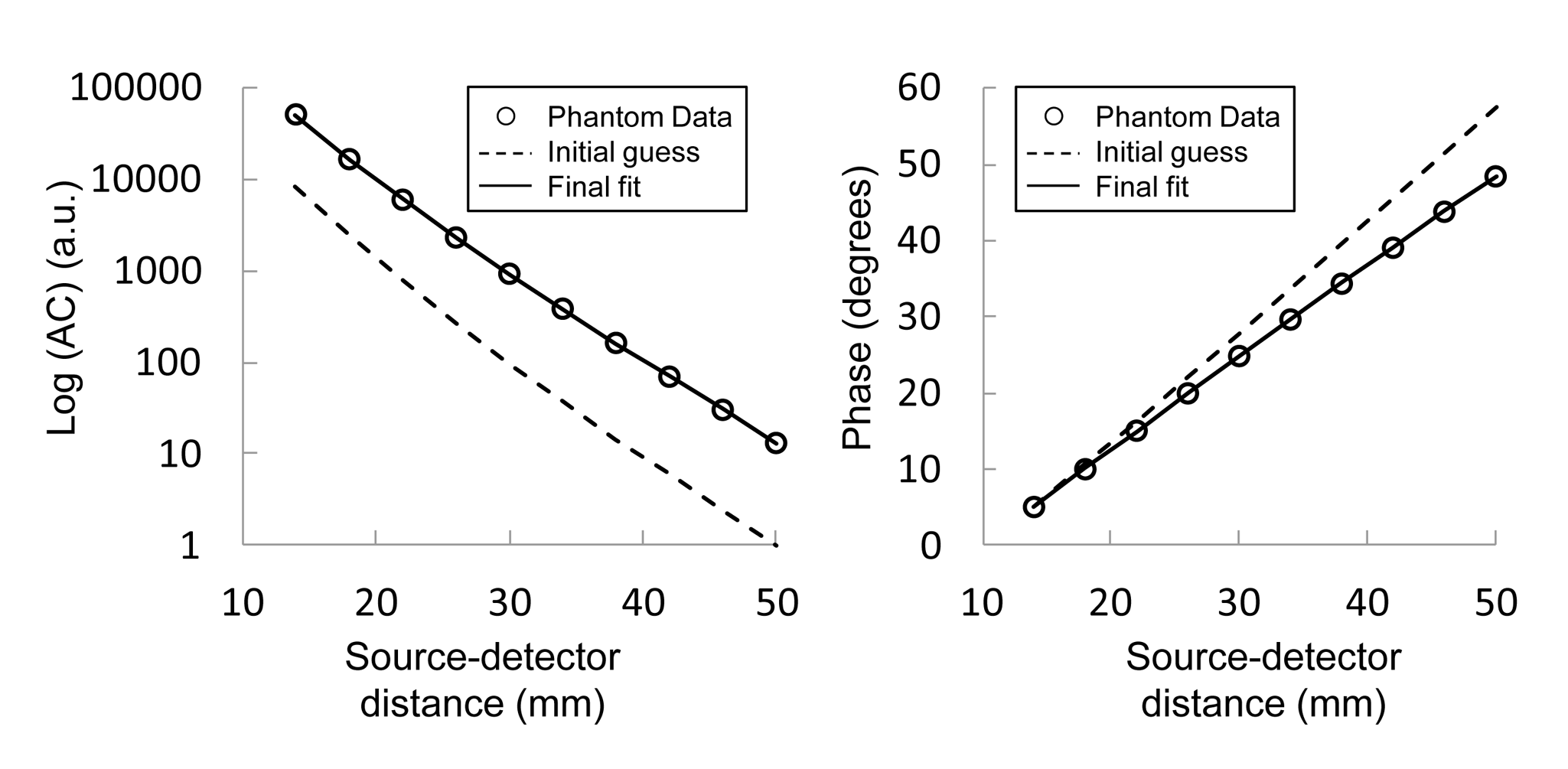

Supplement: Figure S1 — A representative case illustrating the final fit (solid lines) on measurements (circles) of AC attenuation (left panel) and phase shift (right panel) as a function of source-detector distance from the two-layer phantom. Dotted lines illustrate the results yielded by the first iteration of the inversion procedure (using the initial guesses). Final fit was obtained at the 102nd iteration. (TIF) [file pone.0064095.s001.tif]

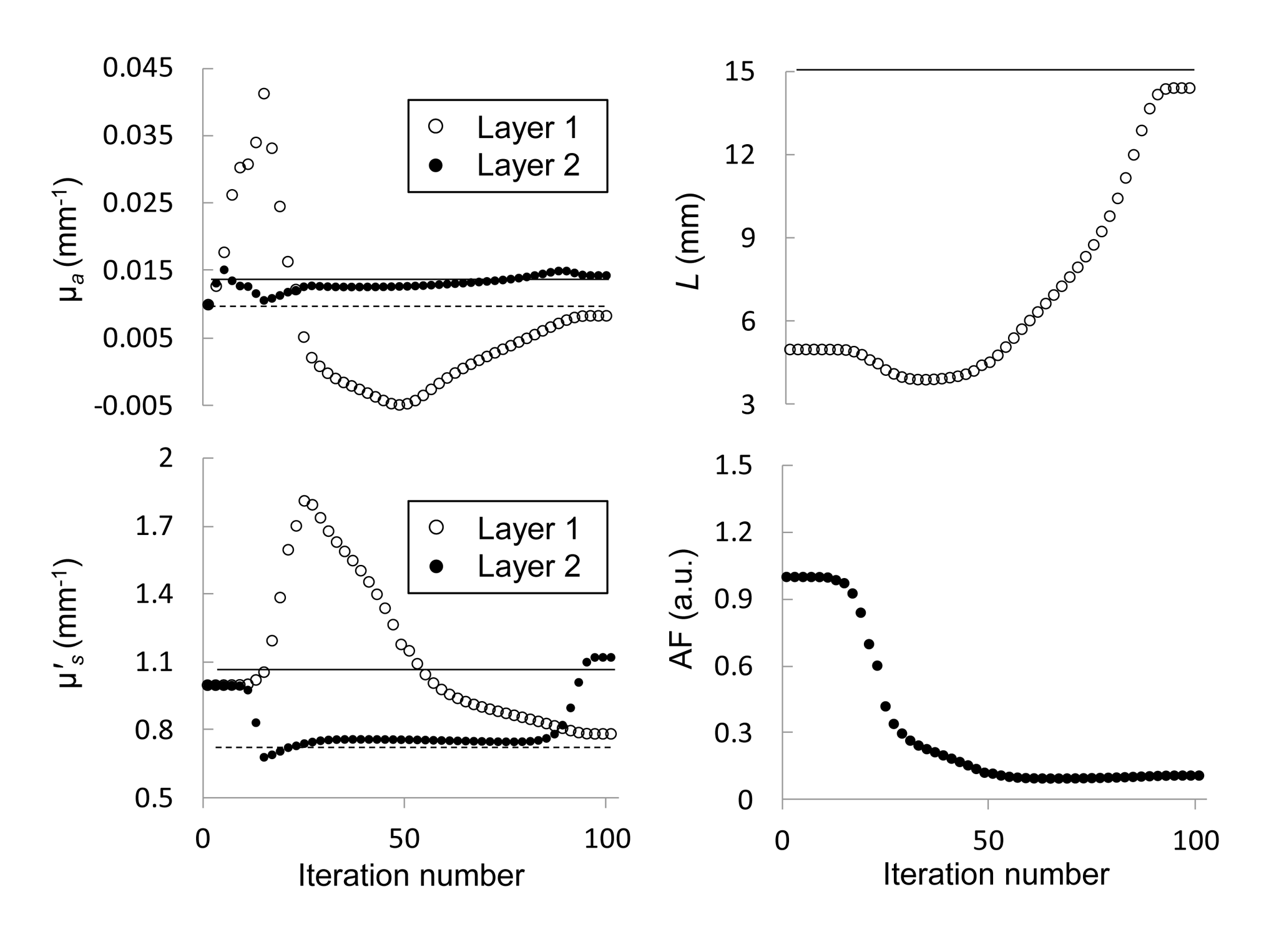

Supplement: Figure S2 — A representative case illustrating the evolution of the six parameters (µ a 1 , µ′ s 1 , L , µ a2 , µ′ s 2 and AF) during the fitting procedure on two-layered phantom data, demonstrating the robustness of the inversion procedure and its insensitivity to initial guesses. Solid and dashed lines represent true values, measured independently in the infinite geometry. (TIF) [file pone.0064095.s002.tif]
